# Clinical Impact of Pelvic Lymph Node Status in Locally Advanced Cervical Cancer Patients Treated by Concurrent Chemoradiation Therapy

**DOI:** 10.31557/APJCP.2021.22.2.491

**Published:** 2021-02

**Authors:** Kanyarat Katanyoo, Thaovalai Thavaramara

**Affiliations:** 1 *Radiation Oncology Unit, Department of Radiology, Faculty of Medicine Vajira Hospital, Navamindradhiraj University, Thailand.*; 2 *Department of Obstetrics and Gynecology, Faculty of Medicine Vajira Hospital, Navamindradhiraj University, Thailand. *

**Keywords:** Cervical cancer, pelvic lymph node, concurrent chemoradiation therapy

## Abstract

**Objective:**

To explore the treatment outcomes of locally advanced cervical cancer (LACC) patients with pelvic lymph node enlargement (PLNE) or stage IIIC1 when compared with no PLNE and unknown PLN status (UNK).

**Materials and Methods:**

Retrospective cohort study was designed by matching with the ratio of 1:4:4 for patients with PLNE, no PLNE and UNK between 2003 and 2017. The main factor which was used to match was clinical staging.

**Results:**

All 360 LACC patients who treated as concurrent chemoradiation therapy (CCRT) were composed of 40 with PLNE, 160 with no PLNE and 160 with UNK. The majority of tumor histology (78.9%) was squamous cell carcinoma and 51.1% were diagnosed in stage IIB. Five-year progression free survival rates of patients with PLNE, no PLNE and UNK were 42.7%, 64.5% and 59.0%, respectively (P = 0.191), and corresponding with 5-year overall survival rates of 57.0%, 66.0% and 61.9% (P = 0.608). Patients with PLNE had local recurrence (LR) at 22.5%, compared with no PLNE at 11.3% and UNK at 11.9%. The most common site of LR for patients with PLNE was PLN with odds ratio of 19.7 when using no PLNE as reference (P < 0.001). There was no statistically significant difference between distant metastasis rates in PLN statuses of patients with PLNE, no PLNE and UNK at 20.6%, 30.0% and 26.3%, respectively.

**Conclusions:**

LACC patients with PLNE had a trend of poorer survival rates than patients with no PLNE, while treatment outcomes of patients with UNK were not inferior to no PLNE.

## Introduction

Stage of disease is the most influential factor for survival outcomes of cervical cancer. The important pitfall of staging system by the International Federation of Gynecology and Obstetrics (FIGO) in 2009 was a neglect of pelvic lymph node (PLN) and para-aortic lymph node (PALN) evaluation (Kim et al., 2009). In 2018, FIGO staging system was revised to use imaging including computed-tomography (CT scan), magnetic resonance imaging (MRI) or fluorine-18-fluorodeoxyglucose positron emission tomography/computed tomography (PET ⁄ CT) and pathological findings to detect these nodes (Bhatla et al., 2018). Therefore, stage IIIC1 and IIIC2 were the additional stages when diseases were detected at PLN and PALN, respectively. However, according to the recommendation of FIGO 2018, staging by imaging is non-mandatory in patients who are inaccessible to the equipment (Bhatla et al., 2019).

Regardless of the FIGO 2009 and 2018, the standard treatment for locally advanced stages (stage IB3, IIA2 to stage IVA), is concurrent chemoradiation therapy (CCRT). The external beam radiation therapy (XRT) is designed to the whole pelvis which covers tumor at cervix, parametrium, pelvic side wall and PLN. As a result, the field size of XRT with the presence of disease at PLN or stage IIIC1 remains the same. Moreover, there is no recommended specific treatment such as increasing radiation doses at this enlarged node or adjuvant chemotherapy for this stage (Bhatla et al., 2019). Some retrospective studies reported the negative impact on survival rates in cases with PLN enlargement (>10 millimeters) from CT scan or MRI (Endo et al., 2014; Parker et al., 2009; Lim et al., 2012).

For the routine practice in Thailand, there are some variations of investigation for staging in cervical cancer. Some patients did not receive any special imaging beyond the clinical examination, especially before launching FIGO 2018. This study was aimed to compare the treatment outcomes of locally advanced cervical cancer (LACC) patients who had PLN enlargement with patients who had no PLN enlargement from CT scan and unknown PLN status in terms of overall survival (OS), progression-free survival (PFS) and pattern of treatment failure. 

## Materials and Methods

*Patients and methods*

A retrospective cohort study for all LACC patients, stage IB3 (IB2 in FIGO 2009) and IIA to stage IVA, who received treatment as CCRT between January 2003 and December 2017 at Faculty of Medicine Vajira Hospital was taken place. After an approval from the Ethics Committee for Research involving Human Subjects of the institution, all medical records were reviewed. LACC patients with squamous cell carcinoma (SCCA) and adenocarcinoma (ADC) as confirmed by tumor histology and received complete treatment by CCRT were included in this study. Patients with human immunodeficiency virus (HIV) infection or received previous surgery were excluded. For the routine practice at Faculty of Medicine Vajira Hospital, clinical staging including physical examination and pelvic examination were performed by radiation oncologists and gynecologic oncologists for all patients. Some patients were procured CT whole abdomen from our Institute or other hospitals before referring to treatment, but some patients did not receive this imaging because of waiting for a long time (1-2 months) to get this investigation. For patients who received CT whole abdomen, para-aortic lymphadenopathy (>10 millimeters) or stage IIIC2 were also removed from this study. All patients who had PLN enlargement (>10 millimeters) were recorded. Patients with normal size of PLN or did not received CT scan underwent a process of review. During that period, there were data of 552 LACC patients who received treatment as CCRT. Of 552 patients, CT whole abdomen was available for 290 patients and 40 of them had only PLN enlargement from official CT report. Due to the limited number of patients with enlarged PLN, matching process with the ratio of 1:4:4 for patients with PLN enlargement, no enlargement and unknown status was attempted in order to increase the power of statistic. Clinical staging (FIGO 2009) was the only factor used for matching in this study. Selection bias was considered to protect with concealed data of treatment outcomes during matching process. Finally, a total of 360 LACC patients were included in this study from these matching technique (40 patients with PLN enlargement, 160 patients with no PLN enlargement and 160 patients with unknown status of PLN). The collecting data were age, tumor histology, total treatment time (TTT), as well as treatment outcomes which including response after completing treatment, rate of local recurrent (LR), rate of distant metastatic (DM), site of disease progression, progression-free survival (PFS) and overall survival (OS). 

*Treatment*

The conventional field of whole pelvis was applied to all patients by two-dimensional technique. The upper border of pelvis field was between fourth and fifth lumbar vertebra which covered common iliac lymph nodes. Dose of XRT per time was 2 Gy with a variation of total dose from 50-60 Gy at cervical tumor. All PLN statuses (PLN enlargement, no PLN enlargement and unknown status of PLN) received the same dose of XRT as 50 Gy for common iliac LN and 54-60 Gy for external and internal iliac LN. High dose-rate (HDR) brachytherapy with a dose of 6.5-7.2 Gy at point A was inserted for four to six times once a week. Platinum-based chemotherapy regimen including cisplatin and carboplatin was used in patients with concurrence with XRT. 

*Statistical analysis*

Data analysis used SPSS statistical software version 22.0 (IBM Corp, Armonk, NY). For continuous data including age and TTT, mean or median were reported as appropriate. Tumor histology, clinical stage using the 2009 FIGO, status of PLN and response of treatment were described by percentage. Chi-square test and Fisher exact test were determined between categorical variables, and odds ratio (OR) with 95% confidence intervals (CIs) were reported. All survival outcomes were analyzed using the Kaplan-Meier method and used log rank test to compare between PLN statuses. Moreover, two pairs of PLN statuses were determined. The first one was between no PLN enlargement and PLN enlargement, while the second one was between no PLN enlargement and unknown PLN status. The Cox proportional hazards models was used to estimate the probably significant prognostic factors and was reported as hazard ratios (HR) with 95% CIs. The statistically significant was considered if p-value <0.05. 

## Results

The mean age of 360 LACC patients was 51.6 **±** 11.2 years. The majority of patients (78.9%) had tumor histology as squamous cell carcinoma. More than half of the patients (51.1%) were diagnosed with stage IIB, when physical and pelvic examinations were used for staging. Although clinical stage was solely used as a factor for PLN status matching, other factors including age and tumor histology were similar. TTT was comparable in patients with no PLN enlargement, PLN enlargement and unknown status of PLN with the means of 58.2 **±** 8.0, 60.8 **±** 7.9 and 59.3**±** 8.3 days, respectively. Baseline characteristics of patients and TTT were shown in Table 1.

Complete response was observed in 338 out of 360 patients or about 94% after CCRT was completed. There was no statistically significant difference between PLN statuses for this treatment outcome (P = 0.774). The similar response rates were observed in patients with ADC and patients with SCCA (90.8% vs 94.7%) (P = 0.277). Clinical staging had a considerable impact on response rate with 90.9%, 100%, 98.4%, 66.7%, 90.4% and 71.4% in stage IB2, IIA, IIB, IIIA, IIIB and IVA, respectively (P < 0.001). 

**Figure 1:**
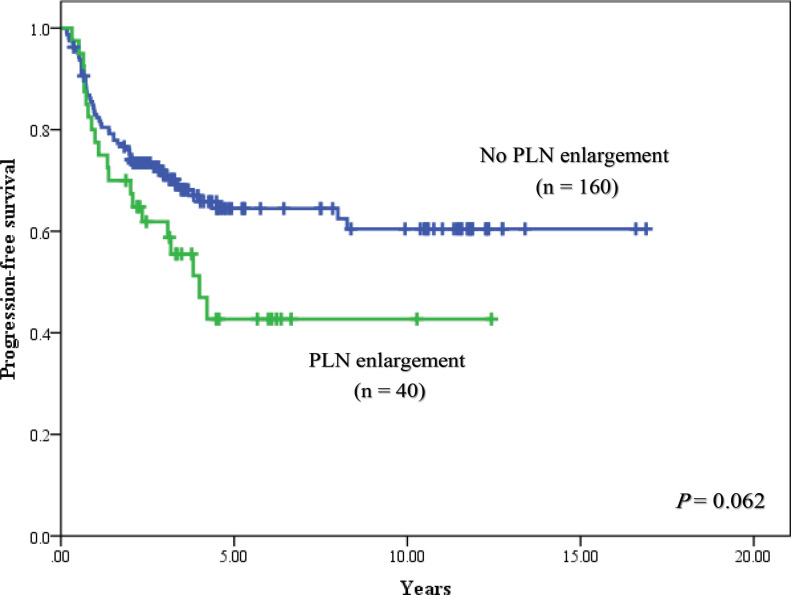
Progression-Free Survival between Patients with No Pelvic Lymph Node (PLN) Enlargement and Pelvic Lymph Node Enlargement

**Figure 2:**
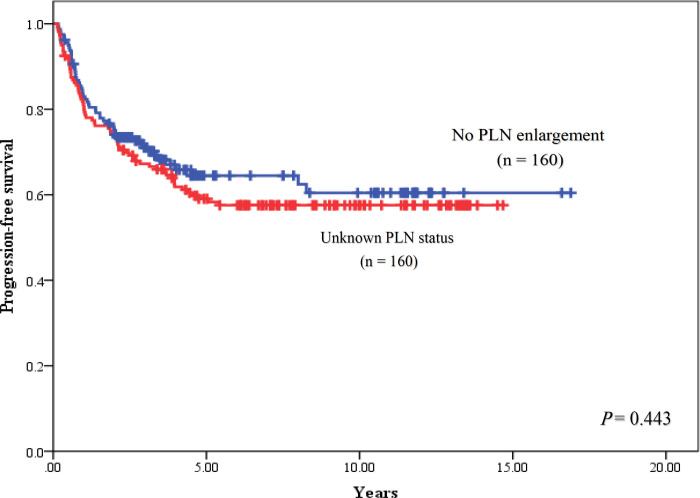
Progression-Free Survival between Patients with Pelvic Lymph Node (PLN) Enlargement and Unknown Status of Pelvic Lymph Node

**Figure 3:**
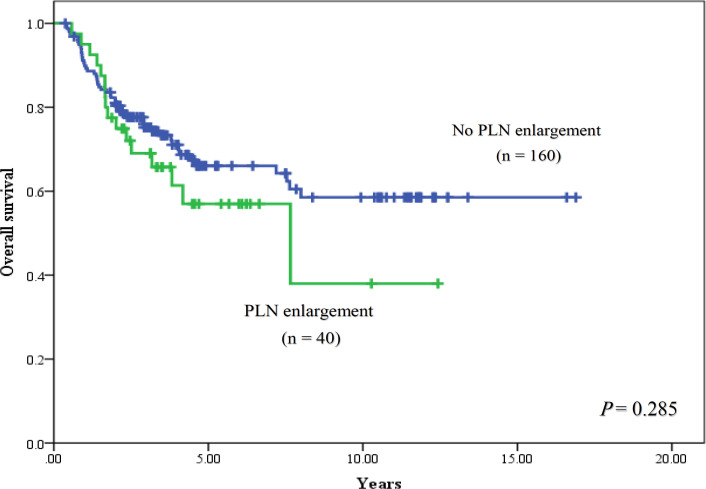
Overall Survival between Patients with no Pelvic Lymph Node (PLN) Enlargement and Pelvic Lymph Node Enlargement

**Figure 4:**
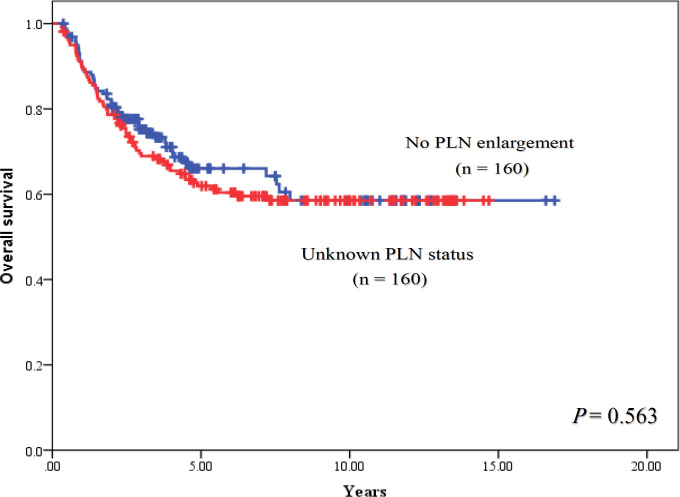
Overall Survival between Patients with no Pelvic Lymph Node Enlargement (PLN) and Unknown Status of Pelvic Lymph Node

The median follow- up time was 7.5 years (range, 2.0-16.9 years). For univariable analysis, 5-year PFS of patients were 50.9% in stage IB2, 100% in stage IIA, 72.7% in stage IIB, 44.4% in stage IIIA, 46.4% in stage IIIB and 0% in stage IVA (P < 0.001), and had the corresponding results of 5-year OS with 62.3% in stage IB2, 100% in stage IIA, 77.8% in stage IIB, 50.0% in stage IIIA, 47.9% in stage IIIB and 0 % in stage IVA, respectively (P<0.001). When PLN status was focused to determine their consequence, 5-year PFS rates of patients with PLN enlargement, without PLN enlargement and unknown PLN status were 42.7%, 64.5% and 59.0%, respectively (P = 0.191). When outcome of patients with no PLN enlargement was used as a reference, HR of patients with enlarged PLN was 1.63 (95% CI = 0.97-2.72) with a trend of statistically significant difference (P = 0.062), while HR of patients with unknown PLN status was 1.15 (95% CI = 0.80-1.66, P = 0.443). The PFS rates were shown in Figure 1 and 2. Five-year OS rates of patients were 57.0% for PLN enlargement group, 66.0% for without enlarged PLN and 61.9% for unknown PLN status. There was also no significant difference between PLN statuses for OS outcomes (P = 0.608) as shown in figure 3 and 4. Median PFS and OS of patients with PLN enlargement were 3.8 years and 4.2 years, respectively. These outcomes had not been reached for patients with negative finding of PLN by CT scan as well as patients with unknown status at this node. For multivariable analysis, when clinical staging was evaluated together with PLN status, there was a statistically significance for staging irrespective of PLN status for survival outcomes (P < 0.001). On the other hand, no statistically significant difference concerning PFS (P = 0.486) and OS (P = 0.785) were shown among patients in the same stage regardless of PLN status. However, interestingly, both PFS and OS rates in stage IIB patients were nearly identical between patients with no PLN enlargement and who with unknown status. The similar result was observed in stage IIIB patients with PLN enlargement and those with unknown status. Survival outcomes comparing between PLN statues in multivariable analysis were demonstrated in Table 2. Results of other stages including Ib2, IIA, IIIA and IVA were not analyzed due to small number of patients. 

Treatment failure was observed in 113 patients (31.4%) including LR in 26 patients (7.2%), DM in 67 patients (18.6%), and both in 20 patients (5.6%). A two-fold increase of local recurrence was shown in patients with PLN enlargement compared with other PLN statuses and had a tendency towards statistical significance (P =0.073).

The most common site of LR for these patients was PLN, which was still existed after complete treatment in 8 out of 40 patients (20.0%). Treatment failure at PLN was also observed in the remaining patients with other PLN statues including 2 patients with no PLN enlargement (1.3%) and 2 patients with unknown PLN status (1.3%). Odds ratio of PLN failure for patients with PLN enlargement was 19.7 when negative finding PLN from CT scan was used as a reference (P<0.001). These results were shown in Table 2. 

**Table 1:** Baseline Characteristic of Patients and Total Treatment Time between Pelvic Lymph Node Statuses

Patients characteristic	Pelvic lymph node statuses (number)		
number (%)	Not enlargement (160)	Enlargement (40)	Unknown status (160)
Age (mean **±** SD)	51.7 **±** 10.9	48.9 **±** 10.3	52.2 **±** 11.6
Tumor histology			
Squamous cell carcinoma	125 (78.1%)	31 (77.5%)	128 (80.0%)
Adenocarcinoma	35 (21.9%)	9 (22.5%)	32 (20.0%)
Clinical staging (FIGO 2009)			
0	4 (2.5%)	1 (2.5%)	6 (3.8%)
IIA	3 (1.9%)	0 (0%)	3 (1.9%)
IIB	84 (52.5%)	20 (50.0 %)	80 (50.0%)
IIIA	4 (2.5%)	1 (2.5%)	1 (0.6%)
IIIB	65 (40.6%)	15 (37.5%)	66 (41.2%)
IVA	0 (0%)	3 (7.5%)	4 (2.5%)
Total treatment time (mean+ SD)	58.2 **±** 8.0	60.8 **±** 7.9	59.3 **±** 8.3

**Table 2:** Comparing Treatment Outcomes between Pelvic Lymph Node Statuses by Cox Proportional Hazard Model Analysis or Odds Ratio

Treatment outcomes(%)	Pelvic lymph node statuses						
	Enlargement (stage IIIC1)	Not enlargement	HR / OR*	p-value	Unknown	HR/ OR*	p-value
			(95% CI)		status	(95% CI)	
CR rate	92.5	95.0	1.54 (0.39-6.09)	0.697	93.1	1.40 (0.55-3.58)	0.637
5-year PFS rate							
All stages	42.7	64.5	1.42 (0.82-2.46)	0.212	59.0	1.14 (0.78-1.65)	0.496
Stage IIB	59.7	73.7	1.54 (0.65-3.64)	0.325	76.6	0.81 (0.43-1.51)	0.503
Stage IIIB	40.6	53.6	1.19 (0.54-2.60)	0.663	40.5	1.35 (0.84-2.18)	0.212
5-year OS rate							
All stages	57.0	66.0	1.25 (0.68-2.27)	0.471	61.9	1.12 (0.76-1.63)	0.571
Stage IIB	77.8	77.4	0.95 (0.32-2.82)	0.931	79.0	0.79 (0.41-1.53)	0.494
Stage IIIB	42.3	54.6	1.3 (0.59-2.87)	0.509	42.7	1.32 (0.82-2.15)	0.257
Rate of LR	22.5	11.3	2.29 (0.94-5.57)	0.073	11.9	1.06 (0.54-2.11)	0.999
Cervix	15.0	11.9	1.84 (0.66-5.14)	0.377	8.8	1.31 (0.49-3.53)	0.791
PLN	20.0	1.3	19.75 (4.01-97.37)	<0.001	1.3	1 (0.14-7.19)	1
Rate of DM	30.0	20.6	1.65 (0.76-3.59)	0.289	26.3	1.37 (0.81-2.30)	0.291
Lung	12.5	11.3	1.13 (0.39-3.24)	0.999	11.3	1 (0.50-2.00)	1
PALN	20.0	10.0	2.25 (0.89-5.71)	0.102	10.0	1 (0.48-2.08)	1

HR, hazard ratio; OR, Odds ratio; CI, confidence interval; PFS, progression-free survival; OS, overall survival; LR, local recurrence; PLN, pelvic lymph node; DM, distant metastasis; PALN, para-aortic lymph node; *HR/ OR and p-value used no pelvic lymph node enlargement as reference

## Discussion

To the best of our knowledge, this study was the first study which explored the treatment results of LACC patients with unknown PLN status comparing with no PLN enlargement and PLN enlargement. For patients in situation of unknown status of this node, their survival outcomes in stage IIB was similar to patients with no PLN enlargement while patients with stage IIIB had comparable treatment outcomes with patients with PLN enlargement. This finding might close some gaps of knowledge about patients whose special investigations were not available or unaffordable. 

PLN, the regional lymph node in cervical cancer, is an influential factor for survival outcomes of cervical cancer patients (Endo et al., 2014; Parker et al., 2009; Lim et al., 2012; Kodaira et al., 2002; Atahan et al., 2007; Cheng et al., 2004). For early stage, Joo et al. explored the association between the ratio of number of PLN diagnosed disease to total number of PLN dissection (PLN ratio) and survival outcomes in early stage of cervical cancer (Joo et al., 2018). They found that high PLN ratio (> 0.40) was poor prognostic factor for treatment outcomes. According to this significant impact, the new stage, IIIC1, was added to the 2018 FIGO staging system. Our study tried to prove the real effect of PLN by control the most important prognostic factors as clinical stage. From the aforementioned process, there was no significant impact of PLN on survival outcomes in LACC patients treated with CCRT. The one reason was all PLN statuses would receive dose of XRT as 50 Gy for common iliac LN and 54-60 Gy for external and internal iliac LN. Therefore, some patients with disease at PLN could get curative treatment from this treatment. Although PFS rate of patients with PLN enlargement was inferior to patients without PLN enlargement at approximately 20% at 5-year, there was not enough power to make a difference by statistical analysis. Previous studies illustrated the statistically significant outcomes from enlarged PLN by imaging. Endo et al. reported poor survival outcomes in patients with PLN enlargement by imaging with HR at 2.25 (95% CI = 1.13-4.48) (Endo et al., 2014), and Paker et al. showed an increasing number of death rate with HR at 2.28 (95% CI=1.04-5.02) (Parker et al., 2009). For pattern of treatment failure, patients with PLN enlargement had significantly PLN failure with OR at 19.75. That was corresponded with study of Jinju et al. (Jinju et al 2019). They reported that recurrence of disease at regional LN was increased with significant difference in patients with PLN enlargement (p<0.001). 

In addition, this study was the first study focusing on the treatment outcomes of patients with unknown PLN status. It is well accepted that cervical cancer was found commonly in underdeveloped and developing countries (Bray et al., 2018), and special investigations prior to treatment were still inaccessible for some patients. Thus, it is undeniable that we should pay more attention to this situation regarding its impact. Interestingly, our results revealed that PFS and OS of patients with clinical stage IIB between no PLN enlargement and unknown status was comparable. While survival outcomes of patients with PLN enlargement and unknown status in clinical stage IIIB were similar. These might explain that patients with unknown PLN status in stage IIIB actually had more incidence of disease at PLN. A retrospective study displayed the incidence of disease at PLN in stage IIIB diagnosed by PET scan was 43% (Singh et al., 2003). Therefore equal prognosis between them and patients with enlarged PLN were shown. These outcomes might suggest that special imaging seem to be more meaningful for predictive survival outcomes of patients with stage IIIB than stage IIB. 

Performance of imaging including sensitivity and specificity is the remarkable factor for detection of PLN. In 2017, a meta-analysis study concerning the ability of CT scan, MRI, Positron Emission Tomography (PET) or PET/CT to determine PLN in cervical was published. They found that PET/CT provided the best performance. The accuracy of CT scan and MRI were approximate values. Sensitivities of CT scan and MRI were 0.57 (95% CI = 0.44-0.69) and 0.54 (95% CI = 0.46-0.61), while specificities were 0.91 (95% CI = 0.88-0.94) and 0.93 (95% CI = 0.91-0.95), respectively (Liu et al., 2017). Therefore, using CT scan or MRI produce the same values of false negative finding around 30% - 50%. As we know that gold standard of diagnosis should be pathological results. For stage IIIC1, two sub-stages are identified as IIIC1r and IIIC1p in case of using radiography and histopathology for PLN diagnosis, respectively. However, a retrospective cohort study explored the method of both diagnostic methods and reported no difference between IIIC1r and IIIC1p (Yang et al., 2020). There was no result of stage IIIC1p in our study due to the exclusion criteria. 

After releasing new staging system as 2018 FIGO system, some studies started to evaluate or verify the new stages, especially for stage IIIC (Yang et al., 2020; Matsuo et al., 2019; Liu et al., 2020; Wright et al., 2019; McComas et al., 2020). Most studies established that cervical cancer patients with disease at PLN or stage IIIC1 had poor prognosis (Matsuo et al., 2019; Liu et al., 2020; McComas et al., 2020).One study reported the better survival outcome of patients with stage IIIC1 than stage IIIB (Wright et al., 2019). However, almost all of the studies established the same result that the significant effect of PLN was being manipulated by tumor size or clinical stage (Matsuo et al., 2019; Liu et al., 2020; Wright et al., 2019; McComas et al., 2020). There was no study focusing on patients with unknown PLN status due to inaccessibility to special imaging from any reasons. 

There were some limitations of this study. Because there was rather small number of patients with enlarged PLN, the matching ratio of them per other PLN statues was one per four. Consequently, the difference of survival outcomes was not shown in statistical analysis, but the potential of statistical significance in outcome of PFS was still observed. Moreover, even though matching process was deliberately done, some biases were still existed unintentionally. The results of CT whole abdomen from various referred hospitals were not standardized by radiologist, so some deviation might be happened. However, the same protocol for diagnosis disease at PLN (>10 millimeters) by CT abdomen was used universal. This limitation might not have affected the results.

Nevertheless, the novel knowledge was generated from this study regarding the value of CT scan to find out patients with stage IIIC1r. The next study that should be conducted in the future is the randomized control trial for proper treatment in stage IIIC1 such as increasing dose of XRT or adjuvant chemotherapy. Additionally, the value of CT scan or other investigations to detect PLN in terms of cost-effectiveness analysis should be also taken into consideration, because all healthcare services have costs. Efficiency is more important than efficacy or effectiveness when there are options in diagnostic methods or treatments. 

To summarize, LACC patients with PLN enlargement had a trend of poorer survival rates than patients with no enlarged PLN, while treatment outcomes of patients with unknown PLN status were not inferior to other patients in the same stage. Using CT scan as a part of staging in the 2018 FIGO system for evaluation PLN status is questionable to gain treatment outcomes.
